# Impact of neonatal resuscitation changes on outcomes of very-low-birth-weight infants

**DOI:** 10.1038/s41598-021-88561-5

**Published:** 2021-04-26

**Authors:** So Jin Yoon, Joohee Lim, Jung Ho Han, Jeong Eun Shin, Ho Seon Eun, Min Soo Park, Kook In Park, Soon Min Lee

**Affiliations:** Department of Pediatrics, Yonsei University College of Medicine, 211 Eonjuro Gangnamgu, Seoul, 06273 Republic of Korea

**Keywords:** Neonatology, Risk factors, Epidemiology

## Abstract

The improvement of delivery room care, according to the 2015 International Consensus, may affect neonatal outcome, especially in very-low-birth-weight infants. We aimed to investigate the current practice of neonatal resuscitation by year and analyze the association with neonatal outcomes. A total of 8142 very-low-birth-weight infants, registered in the Korean Neonatal Network between 2014 and 2017 were included. A significant decreasing trend of intubation (64.5% vs 55.1%, P < 0.0001) and markedly increasing trend of positive pressure ventilation (PPV) (11.5% vs 22.9%, P < 0.0001) were noted. The annual PPV rate differed significantly by gestation (P < 0.0001). The highest level of resuscitation was also shown as an independent risk factor for mortality within 7 days and for bronchopulmonary dysplasia (BPD), intraventricular hemorrhage (IVH), and periventricular leukomalacia. PPV and intubation were associated with significantly decreased risk of mortality and morbidities compared to epinephrine use. When considering association, the incidence of mortality within 7 days, IVH, PVL, and BPD or mortality showed significant differences by combination of year, gestational age, and level of resuscitation. According to updated guidelines, changes in the highest level of resuscitation significantly associated with reducing mortality and morbidities. More meticulous delivery room resuscitation focusing on extreme prematurity is needed.

## Introduction

The majority of very-low-birth-weight infants (VLBWI) require respiratory assistance at birth, although even the most extremely premature infants can breathe or cry at birth. Adequate respiratory support can help improve the aeration of the lungs and circulatory transition^1^. The risk of mortality is the highest immediately after birth, with 25–45% of neonatal mortality occurring during the first 24 h after birth^2^. Delivery room (DR) care for preterm infants is important and is referred to as the golden hour care^3^.

The International Liaison Committee on Resuscitation (ILCOR) was formed in 1992 to identify and review international science and information relevant to cardiopulmonary resuscitation (CPR) and to establish consensus on treatment recommendations. The 2015 International Consensus on neonatal resuscitation was published to identify several unclear and contentious DR resuscitation issues^4^.

The increasing intensity of DR resuscitation has been associated with increasing mortality and neurodevelopmental impairment in VLBWI^5–8^. In a cohort of extremely-low-birth-weight infants (ELBWI) from the Neonatal Research Network centers, infants who received chest compressions and/or epinephrine in the DR (DR-CPR) had increased risk of grade 3–4 intraventricular hemorrhage (IVH), bronchopulmonary dysplasia (BPD), death within 12 h, and neurodevelopmental impairment in survivors at 18 months of corrected age^9^. A recent study by the Canadian Neonatal Network reported that in ELBWI, DR-CPR was associated with higher mortality or neurodevelopmental impairment and lower motor scores at 18–24 months of corrected age^10^. In the Vermont Oxford Network, survival was reported to be 53.8% with DR-CPR compared with 74.9% without DR-CPR, and severe IVH was observed in 15.3% with DR-CPR compared with 4.9% without DR-CPR^7^. Higher intensity of resuscitation was associated with increased risks of mortality, IVH, and periventricular leukomalacia (PVL)^5^.

In Korea, almost all VLBWI (91.7%) require resuscitation at birth, whereas chest compressions and epinephrine administration are required in only 5.4% and 4.1%, respectively. DR-CPR resulted in higher mortality within 7 days and increased incidence of IVH, PVL, and necrotizing enterocolitis (NEC) compared to PPV alone^11^. However, the association of different intensities of resuscitation in the DR and neonatal morbidity according to the updated neonatal resuscitation guidelines has not been elucidated. In this study, we aimed to investigate annual changes in the practice of neonatal resuscitation in Korea according to the new 2015 International Consensus and to analyze the association of the type and nature of resuscitation with short-term outcomes.

## Results

Maternal and neonatal characteristics of 8142 VLBWI according to the year are shown in Table 1. Annual distributions according to sex, gestational age (GA), birth weight, and small-for-gestational-age (SGA) status were not significantly different. The incidence of antenatal steroid use, maternal chorioamnionitis, pregnancy-induced hypertension, maternal diabetes, and cesarean delivery significantly increased according to the year. Surfactant use in the DR increased from 2014 to 2016 but decreased in 2017.

**Table 1:** Demographics and outcome of the very-low-birth-weight infants.

	Total (N = 8142)	2014 (N = 1917)	2015 (N = 2178)	2016 (N = 2134)	2017 (N = 1913)	P value
**Gestational age(weeks)**^**a**^	28.5 ± 2.7	28.4 ± 2.7	28.4 ± 2.7	28.5 ± 2.7	28.5 ± 2.7	0.197
< 25, n (%)	915 (11.2)	213 (11.1)	234 (10.7)	243 (11.4)	225 (11.8)	
25–26^+6^, n (%)	1484 (18.2)	381 (19.9)	429 (19.7)	368 (17.2)	306 (16.0)	
27–28^+6^, n (%)	2044 (25.1)	472 (24.6)	569 (26.1)	515 (24.1)	488 (25.5)	
29–30^+6^, n (%)	2150 (26.4)	473 (24.7)	562 (25.8)	582 (27.3)	533 (27.9)	
≥ 31, n (%)	1549 (19.0)	378 (19.7)	384 (17.6)	426 (20.0)	361 (18.9)	
**Birth weight(g)**^**a**^	1064 ± 287	1060 ± 288	1059 ± 285	1071 ± 288	1066 ± 287	0.556
< 500, n (%)	198 (2.4)	45 (2.3)	57 (2.6)	56 (2.6)	40 (2.1)	
500–749, n (%)	1183 (14.5)	285 (14.9)	311 (14.3)	300 (14.1)	287 (15.0)	
750–999, n (%)	1880 (23.1)	450 (23.5)	497 (22.8)	474 (22.2)	459 (24.0)	
1000–1249, n (%)	2224 (27.3)	502 (26.2)	636 (29.2)	575 (26.9)	511 (26.7)	
1250–1499, n (%)	2657 (32.6)	635 (23.9)	677 (31.1)	729 (34.2)	616 (32.2)	
Male, n (%)	4179 (51.3)	951 (49.6)	1124 (51.6)	1142 (53.5)	962 (50.3)	0.064
Cesarean delivery, n (%)	6335 (77.8)	1424 (74.3)	1694 (77.8)	1693 (79.3)	1524 (79.7)	< 0.0001
Apgar score at 1 min^a^	4.6 ± 2.0	4.6 ± 2.0	4.4 ± 2.0	4.8 ± 2.0	4.6 ± 2.1	< 0.0001
Apgar score at 5 min^a^	6.8 ± 1.9	6.8 ± 1.8	6.7 ± 1.9	6.9 ± 1.8	6.8 ± 1.9	0.006
Rupture of membrane, n (%)	3030 (37.5)	745 (39.2)	787 (36.4)	796 (37.5)	702 (37.0)	0.306
Chorioamnionitis, n (%)	2480 (36.5)	541 (33.7)	644 (35.6)	687 (38.0)	608 (38.6)	0.012
PIH, n (%)	1527 (18.8)	353 (18.4)	392 (18.0)	399 (18.7)	383 (20.0)	< 0.0001
Maternal diabetes, n (%)	661 (8.1)	135 (7.0)	153 (7.0)	180 (8.4)	193 (10.1)	0.002
Prenatal steroid, n (%)	6655 (82.8)	1477 (78.0)	1718 (80.1)	1802 (85.3)	1658 (88.0)	< 0.0001
SGA, n (%)	1356 (16.8)	313 (16.4)	364 (16.8)	352 (16.7)	327 (17.2)	0.931
Surfactant use in DR, n (%)	2775 (56.5)	664 (56.8)	785 (57.7)	735 (57.7)	591 (53.5)	0.139
BPD(≥ moderate), n (%)	2130 (30.4)	484 (29.4)	548 (28.9)	544 (30.0)	554 (33.5)	0.014
IVH (≥ grade 3), n (%)	753 (9.6)	214 (11.6)	196 (9.4)	189 (9.2)	154 (8.4)	0.006
PVL, n (%)	614 (7.9)	158 (8.7)	157 (7.5)	139 (6.8)	160 (8.7)	0.071
NEC, n (%)	563 (7.0)	120 (6.3)	135 (6.2)	166 (7.8)	142 (7.5)	0.093
Sepsis, n (%)	1774 (21.9)	421 (22.0)	453 (20.8)	464 (21.9)	436 (22.9)	0.467
ROP, n (%)	823 (11.8)	196 (11.9)	214 (11.3)	212 (11.8)	201 (12.2)	0.874
Mortality in 7 days, n (%)	536 (6.6)	127 (6.6)	145 (6.7)	152 (7.1)	112 (5.9)	0.443
Mortality, n (%)	1190 (14.6)	279 (14.6)	303 (13.9)	336 (15.7)	272 (14.2)	0.139

*PIH* pregnancy-induced hypertension, *SGA* small for gestational age, *DR* delivery room, *BPD* bronchopulmonary dysplasia, *IVH* intraventricular hemorrhage, *PVL* periventricular leukomalacia, *NEC* necrotizing enterocolitis, *ROP* retinopathy of prematurity, *SD* standard deviation.

^a^Mean ± SD.

**Figure 1:**
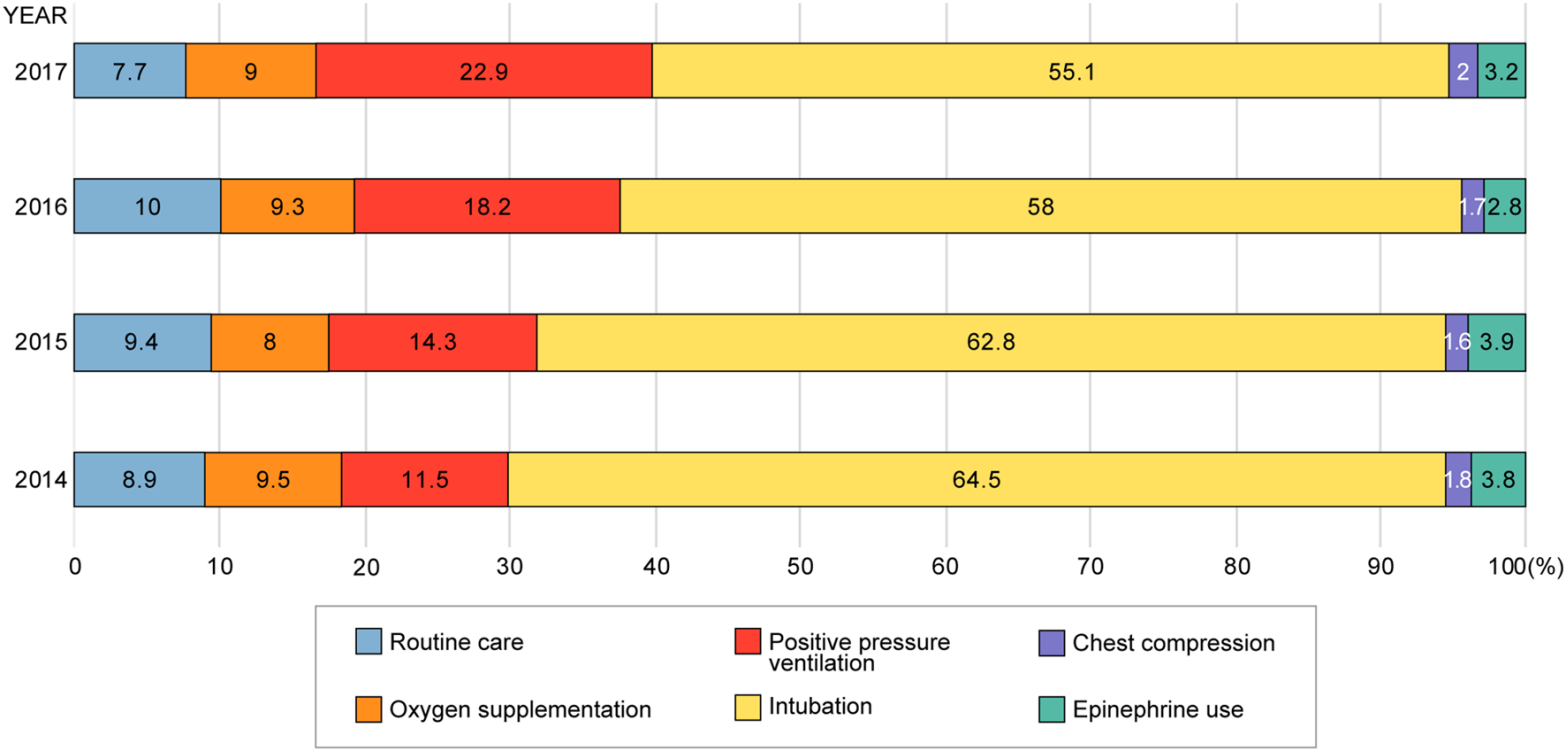
Trends in the highest level of resuscitation in the delivery room. Data are presented as percentage values.

The annual trend for the highest level of resuscitation in the DR is shown in Fig. 1. A total of 735 (9.0%) patients received routine care without resuscitation; 729 (9.0%), only oxygen supplementation; 1359 (16.7%), PPV without intubation, chest compressions, and epinephrine use; 4895 (60.1%), intubation without chest compressions and epinephrine use; 145 (1.8%), chest compressions without epinephrine use; and 279 (3.4%) received epinephrine. According to the year, the incidence of intubation without compressions markedly decreased (64.5% vs 55.1%) (P < 0.0001) and that of PPV gradually increased from 2014 to 2017 (11.5% vs 22.9%) (P < 0.0001).

In multivariate logistic regression analysis (Table 2), the highest level of resuscitation was identified as an independent risk factor for mortality within 7 days. GA, Apgar score at 5 min, maternal chorioamnionitis, antenatal steroid use, and SGA status were also independent risk factors. Especially, PPV (adjusted odds ratio [aOR], 0.27) and endotracheal intubation (aOR, 0.37) were associated with a significantly decreased risk of mortality within 7 days compared to epinephrine use. The highest level of resuscitation was also identified as an independent risk factor for BPD, IVH, and PVL in addition to GA, Apgar score at 5 min, and/or SGA status, and surfactant use. Endotracheal intubation and PPV were associated with a significantly decreased risk of BPD, IVH, and PVL compared to epinephrine use. Overall, the highest level of resuscitation was significantly associated with sepsis (P < 0.0001) and retinopathy of prematurity (ROP) (P = 0.0019); however, the aOR among each level of resuscitation compared to epinephrine use showed a partially significant association.

**Table 2:** Analysis of the risk factors for morbidity in 7 days, BPD, IVH, and PVL using multivariable mixed regression model.

	Mortality in 7 days	BPD	IVH	PVL
**Gestational age (week)**^**a**^				
< 25	20.10(9.30–43.42)	27.36(18.5–40.47)	21.07(11.16–39.75)	1.56(1.02–2.38)
25–26^+6^	7.23(3.39–15.44)	12.23(8.99–16.64)	8.86(4.77–16.44)	1.59(1.09–2.33)
27–28^+6^	3.65(1.70–7.84)	5.37(4.01–7.18)	4.25(2.29–7.86)	1.16(0.80–1.68)
29–30^+6^	2.19(1.00–4.76)	3.12(2.36–4.12)	1.70(0.90–3.20)	1.05(0.73–1.50)
Apgar score at 5 min	0.80(0.75–0.85)	0.84(0.81–0.88)	0.89(0.85–0.94)	0.92(0.87–0.97)
**Highest level of resuscitation**^**b**^				
Routine care	0.17(0.05–0.51)	0.32(0.18–0.56)	0.44(0.21–0.94)	0.46(0.24–0.87)
Oxygen supplementation	0.03(0.004–0.26)	0.26(0.15–0.44)	0.19(0.08–0.46)	0.50(0.28–0.90)
Positive pressure ventilation	0.27(0.14–0.50)	0.37(0.23–0.60)	0.46(0.27–0.78)	0.54(0.33–0.9)
Endotracheal intubation	0.37(0.25–0.55)	0.59(0.38–0.92)	0.61(0.41–0.91)	0.65(0.43–0.99)
Chest compression	0.27(0.13–0.57)	0.75(0.40–1.42)	0.85(0.47–1.57)	1.19(0.65–2.16)
Chorioamnionitis	0.62(0.48–0.79)			
Small for gestational age	1.66(1.16–2.39)	2.56(2.05–3.19)		
Prenatal steroid	0.55(0.42–0.71)			
Surfactant use		1.53(1.17–2.00)		1.96(1.30–2.94)

Only statistically significant variables (P < 0.05) are shown as adjusted odds ratio (95% confidential intervals).

*BPD* bronchopulmonary dysplasia, *IVH* intraventricular hemorrhage, *PVL* periventricular leukomalacia.

^a^Gestational age ≥ 31 weeks is the reference level.

^b^Epinephrine use is the reference level.

**Figure 2:**
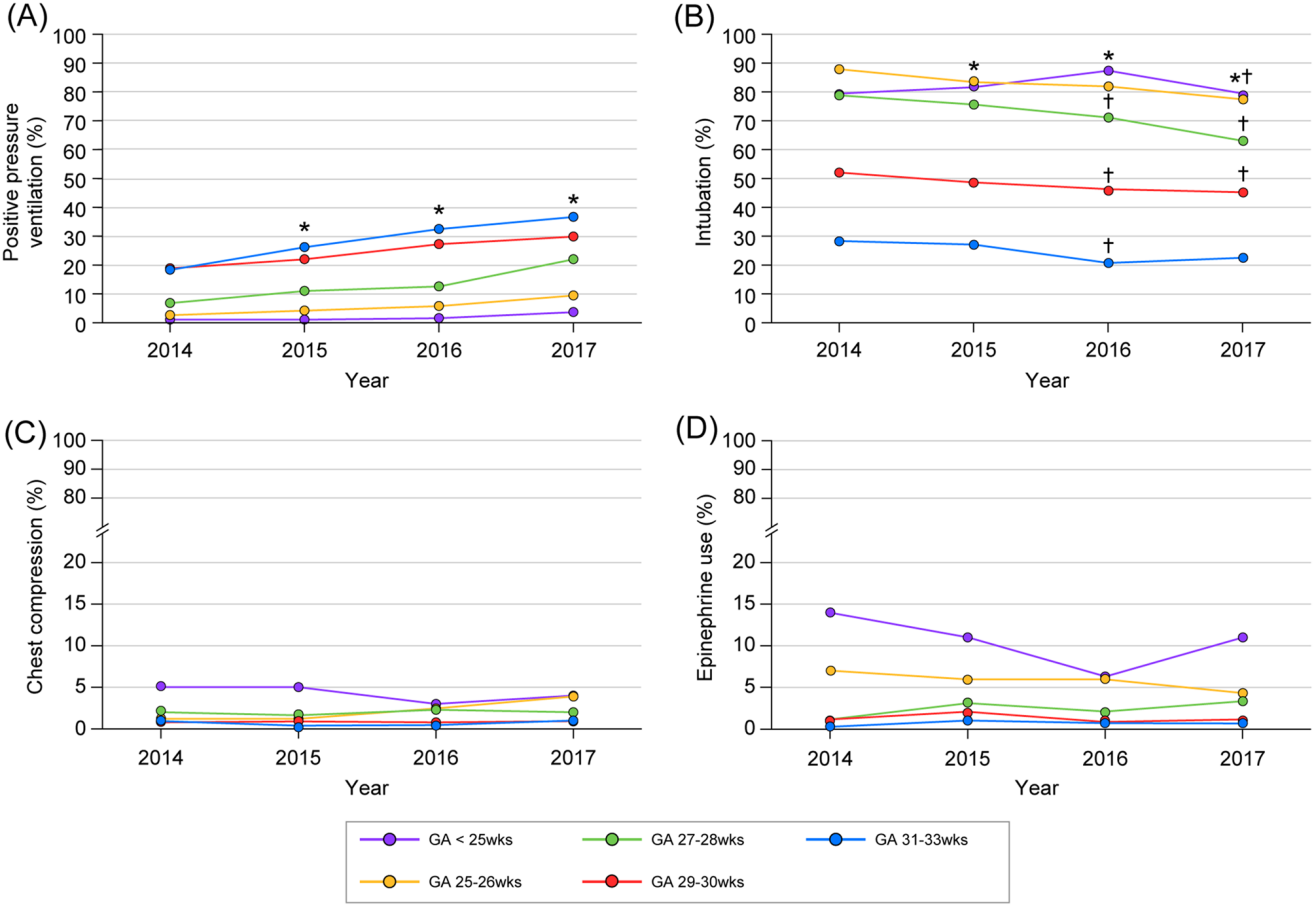
Trends in the highest level of resuscitation in the delivery room stratified by gestational age. The graph shows **(A)** positive pressure ventilation, **(B)** intubation, **(C)** chest compressions, and **(D)** epinephrine use in VLBWI with GA < 25 weeks (purple), 25–26 weeks (yellow), 27–28 weeks (green), 29–30 weeks (red), and 31–33 weeks (blue). *GA* gestational age, *wks* weeks, *VLBWI* very-low-birth-weight infants. *P value < 0.05, compared with rate in 2014. ^†^P value < 0.05, compared with rate in 2014, by GA subgroup analysis.

The serial PPV trends over year differed significantly according to the GA group (P < 0.0001). A generalized estimating equation model revealed that the intubation rate differed according to the GA subgroups and year (P = 0.01). Post-hoc analysis revealed that the intubation rate markedly decreased in 2016 compared to that in 2014, especially in infants with a GA ≥ 27 weeks. This rate also decreased in 2017 compared to that in 2014 among infants with GA ≥ 25 weeks. The incidence of chest compressions and epinephrine use showed significant differences only on stratification according to the GA subgroup but not on stratification according to the year (Fig. 2).

**Figure 3:**
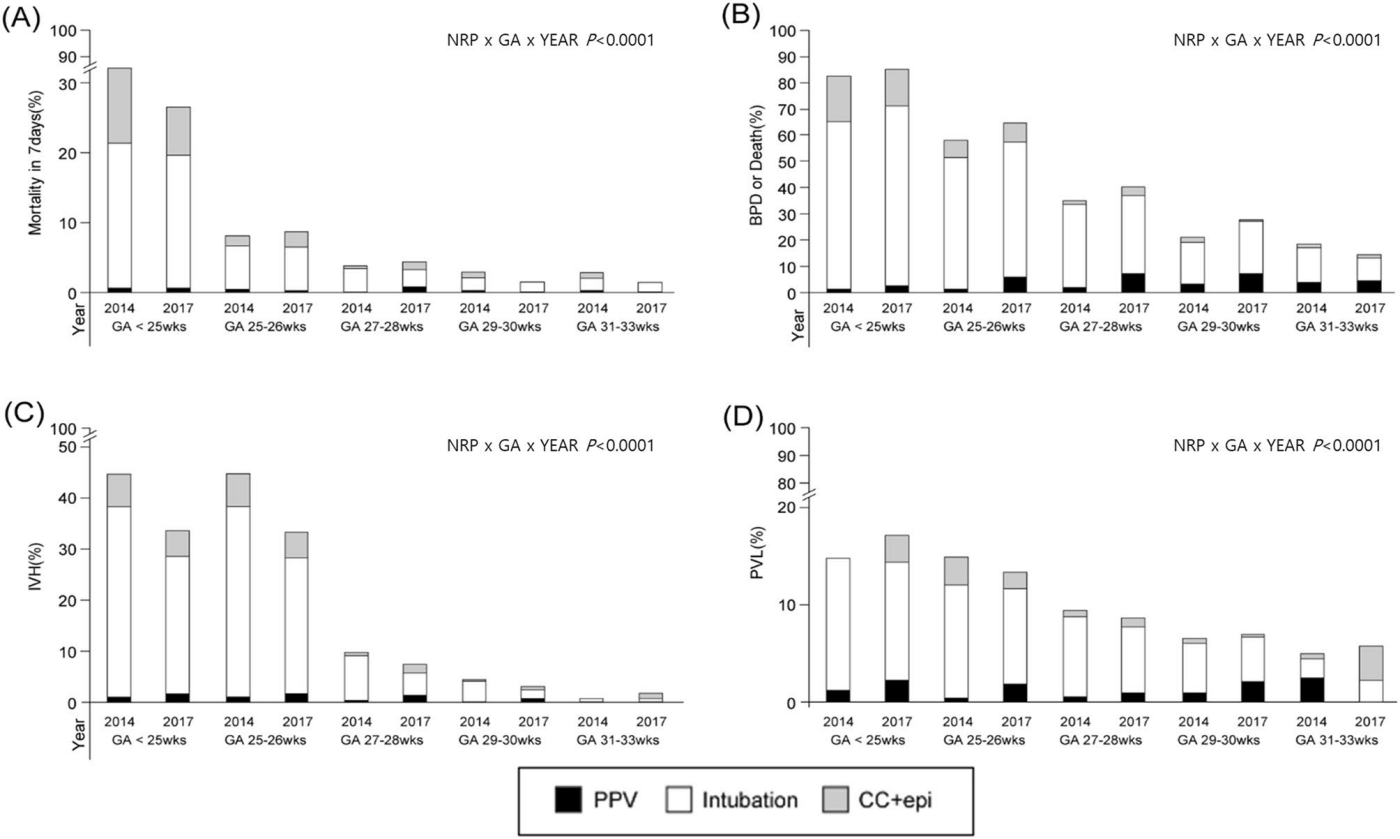
Incidence of neonatal outcome versus gestational age and year according to the highest level of resuscitation in the delivery room. The graph shows **(A)** Mortality in 7 days, **(B)** BPD or death, **(C)** IVH, **(D)** PVL in VLBWI by GA, and year stratified on PPV (black), intubation(white), and CC + epi(gray). *BPD* bronchopulmonary dysplasia, *IVH* intraventricular hemorrhage, *PVL* periventricular leukomalacia, *VLBWI* very-low-birth-weight infants, *GA* gestational age, *wks* weeks, *PPV* positive pressure ventilation, *CC* chest compression, *epi* epinephrine use, *NRP* neonatal resuscitation program.

The association among the outcomes (mortality within 7 days, BPD or mortality, severe IVH, and PVL) according to the level of resuscitation and within effects (GA subgroup and the year of 2014 and 2017 which included before and after changes of guideline) is shown in Fig. 3. Mortality within 7 days, IVH, PVL, combined mortality and BPD showed significant differences when considering the combination effects of the year, GA, and the level of resuscitation. According to the GA subgroup, mortality within 7 days showed significant differences the combination of the level of resuscitation and year, especially in the infants with GA < 25 weeks. IVH showed significant differences the combination of the level of resuscitation and year, especially in the infants with GA 27–28 and 29–30 weeks.

## Discussion

To the best of our knowledge, this is the first study to analyze trends in resuscitation at the DR in VLBWI and the effect on neonatal outcomes, according to the new 2015 International Consensus on neonatal resuscitation. This study showed the admirable progress of trends in DR resuscitation. Furthermore, changes in the practice of the highest resuscitation level especially increasing PPV and decreasing endotracheal intubation were significantly associated with mortality within 7 days and morbidities, such as IVH, PVL and combined mortality with BPD, when considering the effects of gestation and the year before and after the guideline changes. Although the limitation applying the cause and effect directly, this study suggests that improving the quality of resuscitation at the DR may contribute toward the decreased incidence of major morbidities and early mortality. However, continued work to improve resuscitation in the DR at the lower gestational ages will be needed.

The Vermont Oxford Network states that 9.3% of infants weighing 401–500 g and 6% of those weighing 501–1500 g received chest compressions (82.1%) or epinephrine (66.7%).^7^ Our data are consistent with this statement, as 89.1% of the infants in our study received PPV; 71.5%, intubation; 5.4%, chest compressions; and 3.8%, epinephrine. Other studies reported that 2.0% of extremely preterm infants received chest compressions and/or epinephrine in the DR and that the rates of mortality, IVH, and ROP were significantly higher^11^. In the current study, the highest level of resuscitation was an independent risk factor for mortality within 7 days and for IVH, BPD, and PVL.

Through the years, a change in ventilation strategies has been noted, such that proper ventilation with continuous positive airway pressure was originally emphasized rather than initial intubation^12–14^. Noninvasive ventilation was preferred for long-term favorable outcome. In this study, a significantly decreasing trend of intubation without compressions from 2014 to 2017 (76.6% vs 65%) was noted. PPV without intubation rate changed from 11.5% in 2014 to 22.9% in 2017 (P < 0.001). However, this intubation rate is higher than that reported in other neonatal networks, such as 65% compared to 88% for infants with a GA < 28 weeks^15^. The Korean National Health Insurance guideline for reimbursing prophylactic surfactant administration in premature infants of < 30 weeks’ GA at delivery, applied since 2009, could have led to the high incidence of intubation at delivery^16^. In the present study, prophylactic surfactant administration in the DR with elective intubation of premature infants of GA < 30 weeks was practiced in 56.5% of the cases, and there was no significant difference according to the year (P = 0.139). Since 2019, noninvasive surfactant administration has been initiated and applied within insurance coverage; this appears to be a promising strategy to decrease the incidence of intubation at delivery.

Our study showed that the incidence of extensive resuscitation, including chest compressions and/or epinephrine use, has not changed significantly; however, the intubation rate has decreased and the PPV rate has increased yearly. The difference in the intubation rates between 2014 and 2016 was especially prominent. ILCOR published updated neonatal resuscitation guidelines in 2015. The Korean Neonatal Society has held the Neonatal Resuscitation Program (NRP) workshop yearly and introduced the updated information on NRP to many hospital faculties involved in infant delivery. In 2015, the initial use of CPAP rather than intubation and IPPV was recommended, and education of best practices is emphasized in the NRP workshops in Korea. Our results reflected that neonatologists in Korea have applied the updated guidelines in the DR effectively. However, the infants with a higher GA showed apparent changes, and it was still difficult to confirm significant changes in infants with GA < 25 weeks. In the future, the application of the new guidelines should focus on strategies to improve the outcomes for infants with GA < 25 weeks.

Despite the application of several ventilation strategies, BPD, which is the most common chronic respiratory complication in VLBWI, showed increasing incidence due to the increased survival of ELBWI^15^. To address the prevention of BPD in ELBWI, noninvasive ventilator use and noninvasive surfactant administration in the DR are currently being investigated^12,17^. In this study, the annual BPD rate was increasing from 2014 to 2016. The highest level of resuscitation was confirmed as a significant independent risk factor for BPD.

Moreover, there was a significant difference in the risk of BPD between PPV and intubation at the year before and after guideline changes. Further quality improvement projects on resuscitation may lead to a decreasing BPD rate.

Mortality within 7 days of birth showed significant differences according to the GA and decreased in the lower level of resuscitation group. Antenatal steroid exposure has been reported as a protective factor for survival^18–20^. In this study, prenatal steroid use increased from 78.0% in 2014 to 88% in 2017; however, after adjusting for confounding factors, the highest level of resuscitation was also shown as a significant risk factor for mortality within 7 days. (P < 0.0001).

IVH and PVL are important morbidities and may lead to impaired long-term neurodevelopmental outcome. According to the California Perinatal Quality Care Collaborative, a decrease in the IVH rate in infants with a GA of 22–31 weeks was associated with changes in antenatal steroid exposure and DR intubation^21^. In the present study, after adjusting for confounding factors, we found that a higher level of resuscitation increased the IVH or PVL rates however, antenatal steroid use did not have association.

A major strength of our study is that the Korean Neonatal Network included over 70% of VLBWI cared for in neonatal intensive care units in Korea. This information can be extended as a nationwide report on changes in the level of resuscitation and outcomes according to the updated NRP guidelines. The analysis of our study considered the association effects of GA, year and level of resuscitation, and confounding factors such as maternal disease and antenatal care. Therefore, the influence of the level of resuscitation on the neonatal outcomes can be well defined.

Our study carries some limitations. There is a limitation applying the cause and effect directly to explain the highest level of resuscitation in the DR and mortality/morbidities. Although epinephrine is associated with increased mortality and morbidities, PPV and endotracheal intubation do not necessarily be explained as the cause of decreasing mortality and morbidities. The data were collected via electronic case report forms; therefore, some NRP methods may be up for interpretation. Furthermore, the recording of the highest level of resuscitation may be confusing. A wide spectrum of aggressiveness in the management of periviable infants exists across hospitals. Although in 2015, the updated NRP guidelines were revealed and neonatologists and nurses who were in-charge of DR resuscitation were immediately instructed to adhere to these guidelines in Korea, there may be inter-center variation, inter-personal variation, and time gap for the application of the new guidelines. Several factors at delivery room to be related with neonatal outcomes, such as delay cord clamping, initial FiO2, and the use of humidified heated gases, were not considered. In addition, this study cannot consider other respiratory and nutritional factors relating mortality and morbidities in detail during admission.

In conclusion, the highest level of resuscitation was closely related to mortality and morbidities. According to updated guidelines, changes in the resuscitation pattern also significantly contribute to the mortality and morbidities. Efforts to meticulously update the resuscitation guidelines for extreme preterm infants in delivery room will be promising to improve the outcomes.

## Methods

### Subject population

Data for 9032 VLBWI born between 2014 and 2017 in 55–60 Korean Neonatal Network (KNN) centers were prospectively collected using a standardized electronic case report form. The infants with major anomalies and above 34 weeks of GA were excluded. A total of 8142 infants were included in the study. The KNN registry was approved by the institutional review boards of all participating hospitals. Informed consent forms were signed by the parents of the infants during enrollment in the KNN. The present study was performed in accordance with the ethical standards as laid down in the 1964 Declaration of Helsinki and its later amendments and was approved by the KNN data management committee and the Gangnam Severance Hospital IRB (IRB number 3-2013-0020).

### Outcomes and variables

GA was determined according to the best available estimate in weeks and days. GA was further classified as follows: < 25 weeks, 25–26 weeks, 27–28 weeks, 29–30 weeks, and 31–33 weeks. DR resuscitation, including the use of supplemental oxygen, PPV with face mask and bag or T-piece resuscitator without intubation, endotracheal intubation, chest compression, and epinephrine use, was defined as the highest level of resuscitation the infants received. Morbidities were defined as follows: BPD (moderate or severe) was defined as oxygen use at 36 weeks of postmenstrual age^22^; PVL was defined as evidence of PVL on cranial imaging at any time; sepsis was defined by cultures positive for bacteria or fungi and antibiotic therapy ≥ 5 days^23,24^; NEC was defined as clinical and radiographic findings indicating ≥ stage 2^25^; severe IVH was defined as grade 3 or 4 IVH based on cranial imaging performed before 28 days of life^26^; and severe ROP was defined as disease stage ≥ 3 or treatment with retinal ablation surgery or anti-vascular endothelial growth factor drug. For the comparison of outcome before and after change of the guideline, the outcomes at 2014 and 2017 were selected.

### Statistical analysis

We used one-way analysis of variance and χ^2^ test to evaluate bivariate associations between the characteristics and years. Linear regression models were used to determine whether the change in the highest level of resuscitation in the DR was significant. Risk stratification was based on GA or year. A multivariable mixed regression model using the GLIMMIX procedure was used for risk adjustment to predict the factors associated with mortality within 7 days and major morbidities (BPD, severe IVH, sepsis, ROP, and PVL). Variables in the model included GA, mode of delivery, prolonged rupture of membranes, histologic chorioamnionitis, pregnancy-induced hypertension, receipt of antenatal steroid, surfactant use in the DR, SGA status, 5-min Apgar score, and the highest level of resuscitation. The logistic regression models predicted mortality within 7 days and the presence of BPD, IVH, and/or PVL with interaction effects among the different levels of resuscitation, GA, and year. The proportion profile graph was used to show the trends and association among the level of resuscitation, GA, and year. All statistical analyses were performed using SAS version 9.4 (SAS Institute, Cary, North Carolina). P-values < 0.05 were considered statistically significant.

## Data Availability

The dataset analyzed in this study are not publicly available due to the policy of Research of Korea Centers for Disease Control and Prevention. However, datasets are available from the corresponding author on reasonable request.
